# Role of EZH2 in the Growth of Prostate Cancer Stem Cells Isolated from LNCaP Cells

**DOI:** 10.3390/ijms140611981

**Published:** 2013-06-05

**Authors:** Kuiqing Li, Cheng Liu, Bangfen Zhou, Liangkuan Bi, Hai Huang, Tianxin Lin, Kewei Xu

**Affiliations:** 1 Department of Urology, Sun Yat-Sen Memorial Hospital, Sun Yat-Sen University, Guangzhou 510120, China; E-Mails: sykq1920@163.com (K.L.); waynelau2006@163.com (C.L.); Zhoubangfen@163.com (B.Z.); biliangkuan@gmail.com (L.B.); huanghai257@126.com (H.H.); tianxinl@sina.com (T.L.); 2 Key Laboratory of Malignant Tumor Gene Regulation and Target Therapy of Guangdong Higher Education Institutes, Sun Yat-sen University, Guangzhou 510120, China

**Keywords:** prostate cancer stem cells, enhancer of zeste homolog 2, miR-101, cyclin E2

## Abstract

Enhancer of zeste homolog 2 (EZH2) plays a crucial role in embryonic and somatic stem cells for their proliferation and differentiation. However, the roles and underlying mechanisms of EZH2 in prostate cancer stem cells (PCSCs) remain unknown. This study aimed to investigate the effects of EZH2 on PCSCs. PCSCs were isolated from the human prostate cancer cell line LNcap by fluorescence activated cell sorting (FACS). EZH2 expression was compared between PCSCs and non-PCSCs. The association between EZH2 function and PCSC growth was investigated using siRNA-mediated knock-down of EZH2. Cell growth was investigated by MTT, cell cycle and apoptosis of PCSCs were explored by flow cytometric analysis. Finally, the upstream pathway miRNA level was determined via a luciferase reporter assay, and the downstream pathway cycle regulators were examined via reverse transcriptase-polymerase chain reaction. The results showed that LNcap cell line comprised a greater proportion of CD44^+^/CD133^+^ cells by comparison to the PC-3 cell line. EZH2 was up-regulated in PCSCs compared with non-PCSCs. Silence of EZH2 inhibited cell growth and the cell cycle and promoted the progression of apoptosis. Furthermore, EZH2 was a direct target of miR-101 in PCSCs and EZH2’s mRNA levels were inversely correlated with miR-101 expression and cyclin E2 (a cell-cycle regulator) was suppressed by siEZH2. In conclusion, EZH2 is essential for PCSC growth, partly through a negative regulation by miR-101 and positively regulating cyclin E2.

## 1. Introduction

Prostate cancer is one of the most common malignancies in the world. The identification of cells that are involved in prostate carcinogenesis has posed a challenge for many decades [1,2].

Prostate cancer stem cells (PCSCs) have been identified as a low frequency cell population capable of self-renewal and differentiation, which are believed to play an essential role in cancer recurrence following therapeutic intervention [3–5]. PCSCs have been isolated from prostate carcinoma and prostate cancer cell lines using several methods, including flow cytometry for specific cell surface markers, isolation of a side population, and formation of cell spheres [6–9]. Although prostate cancer stem cells have been successfully identified, the molecular pathways regulating their generation and propagation are poorly understood.

Enhancer of zeste homolog 2 (EZH2) plays a central role as a catalytic enzyme involved in histone methylation [10,11]. Indeed, EZH2 can directly methylate the promoters of transcription factors that are essential in sustaining stem cell pluripotency, including Oct-4, Sox-2, and Nanog [12]. Small interfering RNA (siRNA) duplexes targeted against EZH2 lead to early embryonic lethality, meanwhile EZH2 deficient embryonic stem cell lines cannot be established in vitro [13]. Although EZH2 plays a key role during embryogenesis and in a variety of adult stem cells [14–16], little is known regarding the influence of EZH2 on prostate cancer stem cells.

In this study, we investigated the potential involvement of EZH2 in PCSCs. First, we examined the expression level of EZH2 in PCSCs as compared to non-PCSCs. Next, we tested the effects of EZH2 on cell growth, cell cycle and apoptosis by siEZH2. Finally, we explored the mechanisms underlying the effect of EZH2 in PCSCs.

## 2. Results and Discussion

### 2.1. The LNcap Cell Line Contained More CD44^+^/CD133^+^ Cells

**Figure 1:**
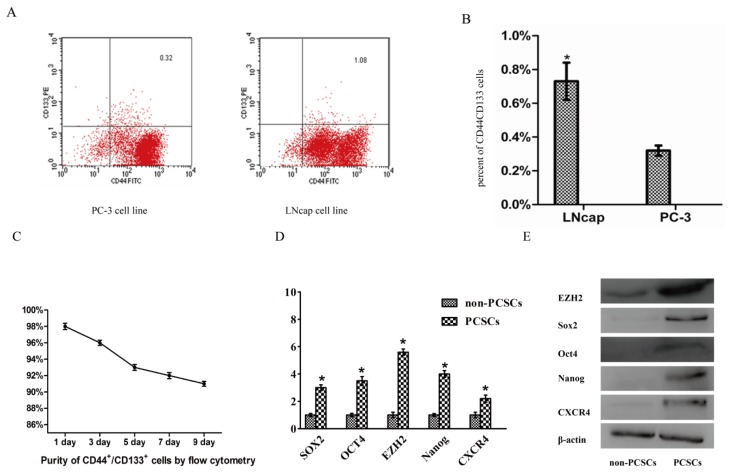
LNcap cell line had more CD44^+^/CD133^+^ cells. EZH2 is up-regulated in prostate cancer stem cells (PCSCs) compared with non-PCSCs. (**A**) Identification of the percent of stem cell like cancer cells in two human prostate cancer cell lines by using flow cytometric analysis; (**B**) Histogram of percent of CD44^+^/CD133^+^ cells (*p < 0.05) in LNcap and PC-3 cell line; (**C**) In all experiments, purity of PCSCs (>90%) was confirmed by flow cytometry. FACS sorted cells were cultured in serum-free medium for 1, 3, 5, 7, 9 days respectively, identification of the percent of CD44^+^/CD133^+^ cells; (**D**) mRNA expression of EZH2 and the putative stem cell markers Oct4, SOX2, Nanog, CXCR4 between PCSCs and non-PCSCs; (**E**) Protein level of of EZH2 and the putative stem cell markers Oct4, SOX2, Nanog, CXCR4 between PCSCs and non-PCSCs.

In order to find a more suitable source of PCSCs, we analyzed the expression of CD44/CD133 in PC-3 and LNcap cell lines using FACS. We found that CD44^+^/CD133^+^ constituted 1.02 ± 0.11% and 0.32 ± 0.03% of LNcap cells and PC-3 cells, respectively (Figure 1A,B). In all experiments, purity of PCSCs (>90%) was confirmed by flow cytometry (Figure 1C).

### 2.2. EZH2 Was Up-Regulated in PCSCs Compared with Non-PCSCs

To confirm expression of previously reported putative stem cell markers in PCSCs, we analyzed mRNA and protein levels of Oct4, SOX2, Nanog, CXCR4 between PCSCs and non-PCSCs. Our results showed that expression of these stem cell markers were up-regulated in PCSCs compared with non-PCSCs. Next, EZH2 expression was measured by qPCR and western blot and results showed EZH2 were up-regulated in PCSCs (Figure 1D,E).

### 2.3. Silencing of EZH2 Suppressed Proliferation and Inhibits Cell Cycle in PCSCs

**Figure 2:**
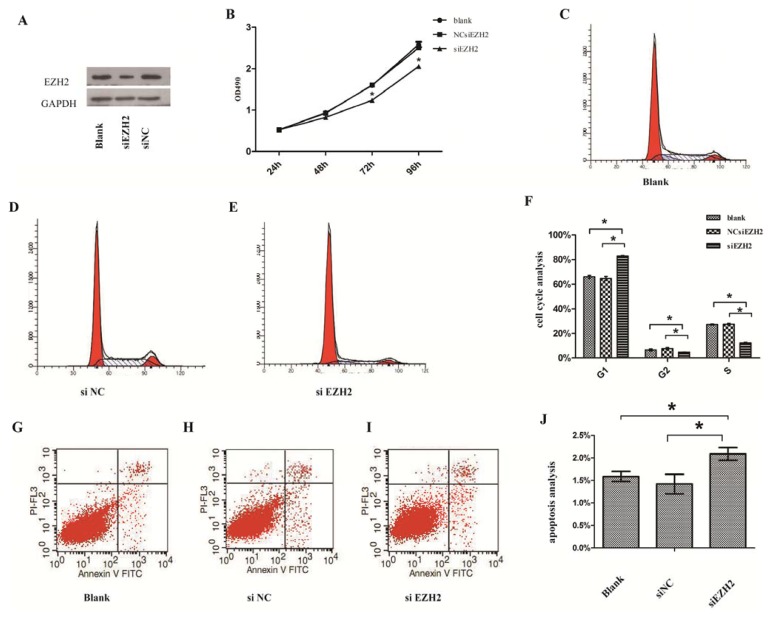
(**A**) Protein levels of EZH2 after PCSCs were transfected with siEZH2 and negative control; (**B**) Cell Proliferation ability in PCSCs by MTT assay; (**C**–**E**) Silence of EZH2 inhibited cell cycle in PCSCs by flow cytometry; (**F**) Histogram of PCSCs cell cycle analysis; (**G**–**I**) Silence of EZH2 promoted the progression of apoptosis. Apoptosis was analyzed by flow cytometry; (**J**) Histogram of PCSCs apoptosis. All data are from 3 separate experiments (*p < 0.05).

To examine whether EZH2 played a tumor-suppressive function, we investigated the effects of EZH2 knockdown on proliferation and cell cycle in PCSCs. Initially, we constructed siEZH2 that can readily block the expression of EZH2 in PCSCs (Figure 2A). Subsequently, the MTT assay demonstrated that siEZH2 reduced cell viability in a time-dependent manner and showed a significant difference compared with controls (Figure 2B). PCSCs transfected with siEZH2 showed an increased percentage of cells in G1 phase but a decreased percentage of cells in S phase compared with controls (Figure 2C–F). Thus, the results revealed that siEZH2 arrested the cell cycle of prostate cancer stem cells.

### 2.4. Silencing of EZH2 Promoted the Progression of Apoptosis

To explore the role of EZH2 in regulating cell apoptosis, siEZH2 or a negative control were transfected into PCSCs. The transfection of siEZH2 was found to promote the progression of PCSCs apoptosis, indicating that EZH2 could inhibit apoptosis of PCSCs (Figure 2G–J).

### 2.5. Cyclin E2 as a Cell-Cycle Regulator Was Suppressed by siEZH2

**Figure 3:**
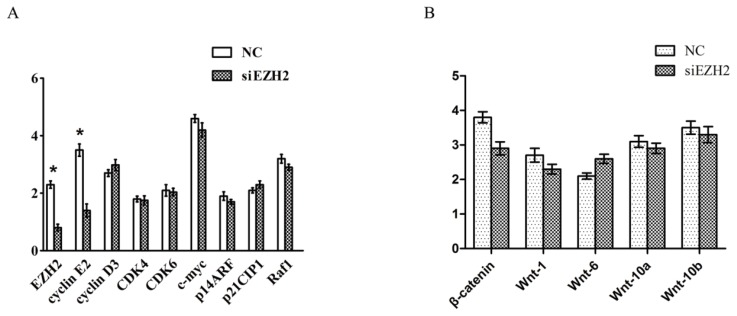
(**A**) RNA levels of 8 cell cycle regulators after PCSCs were transfected with siEZH2 and negative control. Cyclin E2 expression decreased significantly after EZH2 knockdown; (**B**) RNA levels of several embryonic development regulators after PCSCs were transfected with siEZH2 and negative control.

The p53 and pRb pathways are involved in the regulation of cell cycle progression and are frequently deregulated in cancers. To investigate whether these regulators were involved in the growth inhibition by siEZH2, we checked the mRNA levels of 8 regulators by qPCR including cyclin D3 and E2, cyclin-dependent kinase 4 and 6 (CDK4, CDK6), c-myc, p14ARF, p21CIP1, and Raf1(Figure 3A). And we also examined the following embryonic development regulators: β-catenin, Wnt-1, Wnt-6, Wnt-10a and Wnt-10b (Figure 3B). The levels of cyclin E2 decreased after EZH2 knockdown compared with its controls.

### 2.6. EZH2 Was a Direct Target of miR-101 in Prostate Cancer Stem Cells

As previously confirmed, miR-101, miR-124, miR-26a, let-7 can interact with EZH2 in many types of cancer, including glioblastoma, prostate, gastric, breast and bladder cancer [17–26].

**Figure 4:**
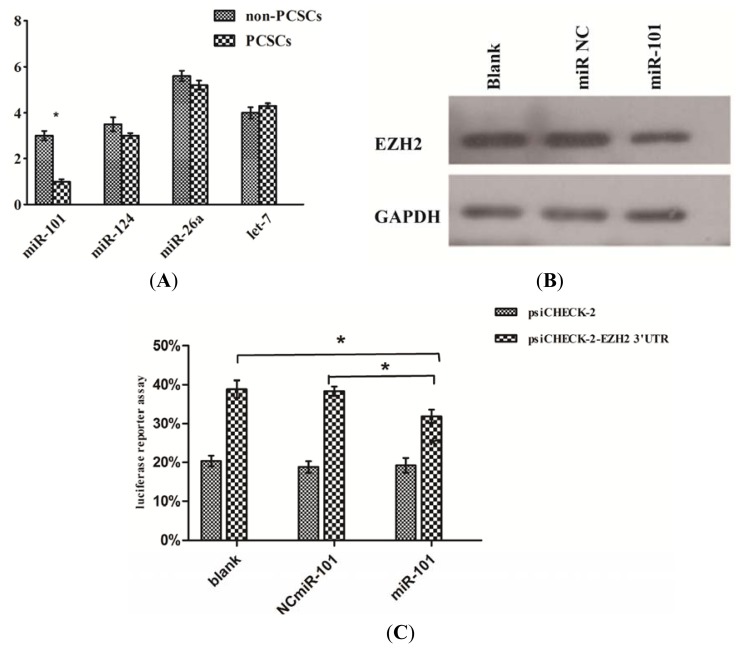
EZH2 was a direct target of miR-101 in prostate cancer stem cells. (**A**) qPCR analysis of several reported aberrant miRNA which could interact with EZH2, down-regulation of miR-101 was confirmed; (**B**) miR-101 mimic could significantly reduced EZH2 expression compared with controls; (**C**) Luciferase reporter assays in PCSCs: compared with controls, miR-101 mimic group showed a significant difference (* *p* < 0.05).

Initially, we examined the expression of miR-101, miR-124, miR-26a, let-7 by qPCR between PCSCs and non-PCSCs, and found that miR-101 expression was significantly down-regulated in PCSCs (Figure 4A).

To validate whether EZH2 was regulated by miR-101 in PCSCs, we transfected miR-101 mimic into PCSCs. As shown, miR101 mimic reduced the EZH2 expression (Figure 4B).

Next, we transfected miR-101 mimic or the negative control into PCSCs along with the psi-CHECK2. By comparison to the negative control, miR-101 mimic significantly suppressed the luciferase activity of the reporter plasmid (Figure 4C). These findings indicated that miR-101 bound to 3′-UTR of EZH2 and repressed EZH2 expression.

### 2.7. Discussion

PCSCs have been identified to be a low frequency cell population with a high proliferative capacity [6,7]. Although a variety of experimental systems have been previously used to study PCSCs [6–8,20,27], the precise mechanisms underlying their generation and propagation had not been demonstrated.

Recent studies have confirmed the important position of EZH2 in maintaining the pluripotency of embryonic stem (ES) cells [13]. Following EZH2 knockout, the level of various genes involved in cell differentiation and embryonic development (MYT1, CNR1, and WNT1) became down-regulated. EZH2 can collaborate with cell growth factors such as OCT-4, SOX-2, and NANOG to maintain stem cell pluripotency, thereby promoting the proliferation and suppressing the differentiation of ES cells [13,28]. Studies of normal tissue stem cells have demonstrated a unique role for EZH2 in stem cell activation [14–16]. EZH2 may play a similarly unique role in cancer stem cells or tumor initiating cells [29].

It’s believed that EZH2 functions mainly through H3K27me3, this kind of methylation leads to down-regulation of target genes. However, recent studies indicated that EZH2 could up-regulate target genes by H3K36me2 [30]. West *et al.* revealed breast tumor-initiating cells displayed high expression of the pluripotency factor Sox2 [31]. Similar with Sox2, higher expression of Oct4 and Nanog were also found in cancer stem cells [32]. Besides, Chang identified increased EZH2 expression in BTICs is linked to enhanced BTICs and high grade breast cancer and OCT4 (OCT4^+^), the detailed mechanism was not illustrated [33]. Recently, Asangani IA discovered that EZH2 could up-regulate target genes by H3K36me2 [30], indicating EZH2 could not only repress target genes, but also up-regulate some genes in another way. The new function of EZH2 might be able to illustrate why EZH2 and the transcription factors including Sox2, OCT4, Nanog are all up-regulated in cancer stem cells. Our study revealed that EZH2 mRNA and protein expression was significantly higher in PCSCs than in matched non-PCSCs, together with the higher expression of stem cell transcription factors. We speculated that EZH2 might up-regulate these factors via H3K36me2 or another similar way. Further study is needed in order to elucidate the relationship between EZH2 and these factors in cancer stem cells.

While investigating the effect of EZH2 on the biological behaviors of PCSCs, we observed that siEZH2 inhibited cell growth and G1/S arrest, and induced apoptosis of PCSCs. These data indicated that EZH2 was essential for PCSCs growth. However, the mechanisms by which EZH2 influenced cell growth were still poorly defined. We checked the expression of important regulators, including p53, pRb, and Wnt pathways, and found that knockdown of EZH2 can down-regulate cyclin E2 expression, which can then regulate G_1_/S checkpoint as an oncogene [34,35]. Additionally, we did not observe a significant reduction in the expression of any other genes which had shown significant differences following EZH2 knockdown. We believe that by down-regulating cyclin E2, siEZH2 can arrest the G_1_/S stage which, in turn, suppressed PCSCs growth.

Aberrant expressions of miRNAs have been reported in regulation of EZH2. Down-regulation of miR-101, miR-124, miR-26a, let-7 have been reported in leading to EZH2 over-expression in various kinds of cancer [17–26]. Additionally, these miRNAs have been shown to be implicated in cancer-related processes such as cell growth, invasion, and apoptosis [17–26]. We speculate that some of these miRNAs may regulate EZH2 expression in PCSCs. We examined the expression of miR-101, miR-124, miR-26a, let-7 between PCSCs and non-PCSCs, and found that miR-101 expression was inversely correlated with EZH2’s mRNA level. And PCSCs transfected with miR-101 mimic showed a dramatic decrease in EZH2 protein level. Furthermore, we found that activity of the luciferase reporter with EZH2 3′-UTR was significantly inhibited in PCSCs transfected with miR-101 mimic compared with those transfected with negative and blank controls. These findings provided strong evidence that miR-101 can down-regulate EZH2 expression by directly targeting the 3′-UTR of EZH2 mRNA.

## 3. Experimental Section

### 3.1. Cell Culture

LNCaP and PC-3 human prostate cancer cells were purchased from the American Type Culture Collection (ATCC, Manassas, VA, USA). PC-3 and LNcap cell lines were cultured at 37 °C with 5% CO_2_ atmosphere in RPMI-1640, which contained 10% fetal bovine serum (FBS) with 100 IU/mL penicillin and 100 μg/mL streptomycin. Prostate cancer stem cells sorted by fluorescence activated cell sorting (FACS) were cultured as spheres in a suspension culture system as previously described [36].

### 3.2. Fluorescence Activated Cell Sorting

To find an efficient method for sorting prostate cancer stem cells from prostate cancer cell lines, we measured the expression of the molecular marker CD44^+^/CD133^+^ using anti-CD44-FITC, anti-CD133-phycoerythrin (PE), their corresponding isotype control antibodies (mouse IgG1-FITC and IgG1-PE (Miltenyi Biotec Inc, Bergisch Gladbach, Germany)), and a fluorescence activated cell sorter (Becton Dickinson, Franklin Lakes, NJ, USA). Cells were harvested, gently disaggregated to a single cell suspension, and stained according to the manufacturer’s protocol.

### 3.3. RT-PCR Analysis of mRNAs and miRNA Expression

Total RNA was extracted using the Trizol reagent (Invitrogen, Carlsbad, CA, USA) and reversely transcribed using ImProm-II Reverse Transcription System (Promega, Madison, WI, USA) to quantify the mRNA levels of Oct4, SOX2, Nanog, CXCR4, EZH2 and cell-cycle regulators, primers were listed in the supplement material (See Table S1). RT-PCR was performed using SYBR Green PCR master mix (Applied Biosystems, Carlsbad, CA, USA) on an ABI 7500HT system. All RT-PCRs were performed in triplicates.

### 3.4. Western Blotting

Cell lysates were separated by 10% SDS-PAGE, and electrophoretically transferred to a polyvinylidene difluoride (PVDF) membrane (Millipore Corporation, Billerica, MA, USA). Then the membrane was incubated with mouse monoclonal antibody against human EZH2 (Cell Signaling Technology, Danvers, MA, USA) followed by horseradish peroxidase (HRP)-labeled goat-antimouse IgG (Cell Signaling Technology, Danvers, MA, USA) and detected by chemiluminescence. Glyceraldehyde-3-phosphate dehydrogenase (GAPDH) was used as a protein loading control. The intensity of protein fragments was quantified with the Quantity One software (Bio-Rad Laboratories, Hercules, CA, USA).

### 3.5. Cell Proliferation Assay

3-(4,5-dimethylthiazol-2-yl)-2,5-diphenyltetrazolium bromide (MTT) substrate (Sigma, St. Louis, MO, USA) was used to assay cell proliferation according to the manufacturer’s instructions. Briefly, each group of cells was seeded at 1 × 10^3^ per well in 96-well plates. Then the MTT reagent (Sigma, St. Louis, MO, USA) at a concentration of 5 mg/mL was added to the maintenance cell medium at different time points (24 h, 48 h, 72 h and 96 h) and incubated at 37 °C for an additional 4 h. The reaction was terminated with 150 μL dimethylsulfoxide per well. The cells were lysed for 15 min and the plates were gently shaken for 5 min. Absorbance values were determined using a microplate reader (Multiscan MK3, Thermo Fisher Scientific, Waltham, MA, USA) at 490 nm.

### 3.6. Cell Cycle Analysis

Cell cycle analysis was performed to evaluate the impact of EZH2 on PCSCs proliferation. Briefly, cells transfected with siEZH2 or controls were washed twice with PBS and fixed overnight with 70% ethanol at −20 °C. Cells were washed with PBS and incubated for 30 min at 37 °C in PBS containing 0.05 mg/mL PI, 1 mmol EDTA, 0.1% Triton x-100, and 1 mg/mL RNase A. Finally, the stained cells were analyzed using flow cytometry.

### 3.7. Apoptosis Assays

The Annexin V Apoptosis Detection Kit (BD bioscience, Franklin Lakes, NJ, USA) combined with flow cytometry was used to observe the effect of siEZH2 on the induction of apoptosis in PCSCs. After incubation with siEZH2, 1 × 10^5^ PCSCs were harvested. Next, cells were washed with PBS and resuspended in 200 μL of binding buffer. After the addition of 5 μL Annexin V conjugate for 10 min of incubation, the samples were resuspended in 200 μL binding buffer and 5 μL propidium iodide (PI). Finally, samples were analyzed using the MACS Quant^®^ analyzer (Miltenyi Biotec Inc, Bergisch Gladbach, Germany). Annexin V-positive cells were designated as apoptotic cells. The data were analyzed using FlowJo software (Tree Star, Inc, Ashland, OR, USA).

### 3.8. Luciferase Reporter Assay

To examine whether miRNA could directly interact with the 3′-UTR of EZH2, we created luciferase reporter plasmids by cloning 3′-UTR of EZH2 into a psi-CHECK2. Luciferase reporter was constructed by cloning 3′-untranslated regions (3′-UTR) of EZH2 mRNA into the XbaI-site of psi-CHECK2 (Promega, Madison, WI, USA). PCSCs were transfected with psi-CHECK2 plasmid by Lipofectamine 2000 and then cotransfected with miR-101 mimic or a negative control. After 48 h, luciferase assays were performed using the Dual-Luciferase assay system (Promega, Madison, WI, USA). For each sample, firefly luciferase activity was normalized to renilla luciferase activity.

### 3.9. Transient Transfection

Cells (1 × 10^6^) were plated in a low-attachment six-well plate in SFM without antibiotics. The EZH2 siRNA, or a negative control oligonucleotide, was transfected into cells with lipofectamine 2000 (Invitrogen, Carlsbad, CA, USA), according to the manufacturer’s instruction. The concentration of EZH2 siRNA was 100 μm. After 6 h of incubation in the CO_2_ incubator, the medium containing siRNA-lipofectamine 2000 complexes was replaced fresh SFM and the cells were cultured for further experiments. The specific siRNA sequence was as follows: 5′-GAAUGGAAACAGCGAAGGA-3′. A non-specific control siRNA was used as a control. The efficiency of RNA interference was monitored by fluorescence microscopy and checked by RT-PCR and Western blot analysis. The specific miR-101 sequence was as follows: UACAGUACUGUGAUAACUGAA. A non-specific control miRNA was used as a control.

### 3.10. Statistical Analysis

Statistical analysis was performed using SPSS software 18.0 [37]. Values were presented as mean ± standard deviation. Differences were assessed by Student’s *T* test or analysis of Variance (ANOVA). A *p* value of <0.05 was considered to be statistically significant.

## 4. Conclusions

In summary, EZH2 is essential for PCSCs growth, partly through regulating cyclin E2, and EZH2 expression is regulated by miR-101. Considering the role of EZH2 in PCSCs and the relationship between EZH2 and miR-101, introduction of miR-101 to silence EZH2 could be a potential therapeutic strategy for prostate cancer.

## Supplementary Information
