# Supplementary material for: Role of EZH2 in the Growth of Prostate Cancer Stem Cells Isolated from LNCaP Cells
Source: Int J Mol Sci. 2013 Jun 5;14(6):11981–93. doi: 10.3390/ijms140611981 (PMC3709767; doi:10.3390/ijms140611981)
Supplement: Supplementary file 1 [file ijms-14-11981-s001.pdf]

# Supplementary Information

**Table S1.** List of primers for PCR.

| Target gene | Forward primer (5'→3') | Reverse primer (5'→3') |
|---|---|---|
| EZH2 | CCAAGAGAGCCATCCAGACT | CGATGCCGACATACTTCAGG |
| CCNE2 | AGGTTTGGAGTGGGACAGTA | ATTGCACACTGGTGACAACT |
| CCND3 | ATACCTTTGCCATGTACCCG | TGGCTGTGACATCTGTAGGA |
| CDK4 | ATGTTGTCCGGCTGATGGA | GTGAACGATGCAATTGGCA |
| CDK6 | GTCATTCAAGGACCACCAAA | GGCAGGTAAGAATGGCTATC |
| AR | CCTGAGATTGAGGTGCTCTT | TCCTAGGCAACAGAAGTGTC |
| Raf1 | CCAGTCGGATGTCTACTCCT | AGCAGCTCAATGGAAGACAG |
| P21C1P1 | CTTCAGTACCCTCTCAGCTC | CTGAGGTAGAACTAGGGTGC |
| Wnt-10b | GAATCCACAACAACAGGGTG | AAGTAGACCAGCTCTCCTGA |
| β-catenin | GCATCAAGGGAGACACCA | TGACCTAACTAAAGCACCAGA |
| P14ARf | CAGAGGCAGTAACCATGCC | TCAGAGCCTCTCTGGTTCTT |
| c-myc | ACACATCAGCACAACTACGC | CCTCTTGACATTCTCCTCGGT |
| wnt1 | TGCCTCTCTTCTTCCCCTTT | CCCTGTAACCTCCTGCTTCA |
| wnt6 | AGCGTGCAGCTCGAAGAGAA | TTTCCAGAGCCCTGGGAGTT |
| wnt10a | AACCCCACCACTCACTTCT | GGTGAAAGGATGGAGACAGA |
| SOX2 | CACCTACAGCATGTCCTACTC | CATGCTGTTTCTTACTCTCCTC |
| Nanog | TGCCTCACACGGAGACTGTC | TGCTATTCTTCGGCCAGTTG |
| CXCR4 | ACTACACCGAGGAAATGGGCT | CCCACAATGCCAGTTAAGAAGA |
| OCT4 | GACAACAATGAAAATCTTCAGGAGA | CTGGCGCCGGTTACAGAACCA |

© 2013 by the authors; licensee MDPI, Basel, Switzerland. This article is an open access article distributed under the terms and conditions of the Creative Commons Attribution license (http://creativecommons.org/licenses/by/3.0/).
